# Method for Whole Mount Antibody Staining in Chick

**DOI:** 10.3791/956

**Published:** 2009-02-02

**Authors:** Delphine Psychoyos, Richard Finnell

**Affiliations:** Institute of Biosciences and Technology, Center for Environmental and Genetic Medicine, Texas A&M Health Science Center

## Abstract

The chick embryo is a valuable tool in the study of early embryonic development. Its transparency, accessibility and ease of manipulation, make it an ideal tool for studying  antibody expression in developing brain, neural tube and somite. This video demonstrates the different steps in whole-mount antibody staining using HRP conjugated secondary antibodies; First, the embryo is dissected from the egg and fixed in paraformaldehyde. Second, endogenous peroxidase is inactivated; The embryo is then exposed to primary antibody. After several washes, the embryo is incubated with  secondary antibody conjugated to HRP. Peroxidase activity is revealed using reaction with diaminobenzidine substrate. Finally, the embryo is fixed and processed for photography and sectioning. The advantage of this method over the use of fluorescent antibodies is that embryos can be processed for wax sectioning, thus enabling the study of antigen sites in cross section. This method was originally introduced by Jane Dodd and Tom Jessell ^1^.

**Figure:**
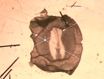


## Protocol

### I. Schematic Overview:

This video demonstrates the different steps in whole mount immunohistochemistry in chick embryo. First, the embryo is fixed in PFA [IHC1]. Then, endogenous peroxidase activity is quenched [IHC2]. The embryo is then incubated in primary antibody [IHC3]. After several washes, the embryo is incubated in secondary antibody [IHC4]; Color reaction is revealed using DAB [IHC5] and antibody staining appears orange [IHC6].


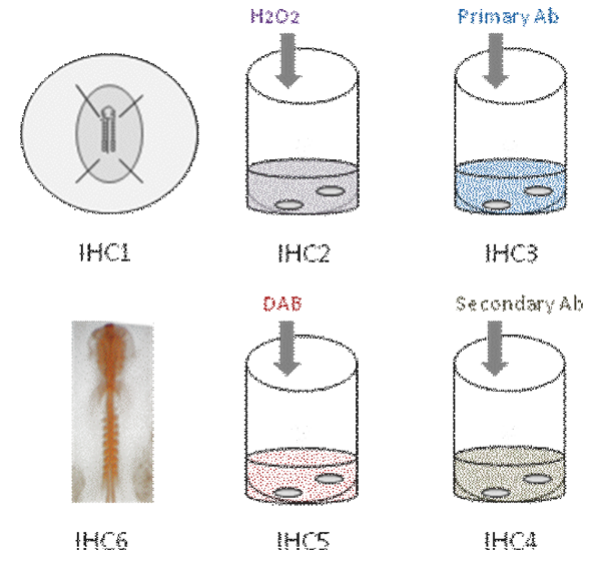


### Part 1: Fixing the embryos

1. To perform whole mount immunohistochemistry on chick embryos, first open an egg by tapping the shell with forceps and removing pieces of the shell.
2. Remove the thick albumin with forceps, and tilt the yolk sac with coarse forceps so that the embryo faces upwards.
3. Using fine scissors, cut a square of yolk sac around the embryo. Remove the embryo from the yolk with a spoon, and place in a dish containing PBS.
4. Under a dissection microscope, carefully remove the membranes and yolk and the embryo transfer to a fresh dish containing PBS.
5. Pin the embryo down with forceps and insect pins, and aspirate the PBS. Replace this with 4% PFA in PBS, and allow the embryo to fix 1h at RT.

### Part 2: Preparing embryos for antibody step

1. To minimize potential microbial contamination, use ddH_2_O in all following steps.
2. After the embryo is fixed, aspirate the fixative solution and dispose properly as chemical waste. Fill the dish with PBS.
3. next, using a microdissection knife, cut a square around embryo to remove extraembryonic membranes. Remove insect pins with forceps.
4. After the pins have been removed, use a blunt end Pasteur pipette to transfer the embryo to a scintillation vial containing with PBT (PBS pH 7.4, 0.5% Triton X).
5. Wash the embryo with PBT, 3 times for 10 minutes each.
6. Remove PBT from the scintillation vial, and replace with PBTx containing 0.3% H_2_O_2_ in order to inactivate potential endogenous peroxidase.
7. Incubate for 2 hr at RT on nutator.
8. Wash embryo with PBT, 3 times for 10 minutes each, then 3 times for 30 minutes each.

### Part 3: Antibody incubation

1. To stain the embryo with antibody, start by washing the embryo for one hour in blocking buffer or 1% BSA/1% NGS/PBT (we use NGS because the secondary antibody is raised in goat). Incubate on nutator for one hour at RT.
2. Dilute the primary antibody 1:1 in blocking buffer, and incubate embryo in this solution for 2 days  at 4°C on nutator. Primary antibody dilution factor depends on the choice of primary antibody. Here, we use an antibody from the Developmental Studies Hybridoma Bank, which is provided as supernatent . We dilute it 1:1 in blocking buffer.
3. next, perform 3 10-minute washes in PBT, followed by 3 1-hour washes in PBT.
4. After washing, dilute the secondary antibody 1:2500 in blocking buffer (in this case peroxidase conjugated-goat anti-mouse IgG (H+L), and incubate embryo in this solution O/N at 4°C on nutator.
5. Perform 3 10-minute washes in PBT, followed by 3 1-hour washes in PBT.

### Part 3: Color reaction

1. To develop the color reaction of the stained embryo, first perform 2 20-minute washes in Tris buffer (100mM Tris HCl, pH 7.4).
2. Meanwhile dissolve DAB substrate (3,3’-diaminobenzidine tetrahydrochloride) in Tris buffer at 500μg/ml under fume hood; keep solution in dark, on ice.
3. Remove last Tris buffer wash from the vial containing embryo and replace with 5ml DAB solution from step 3.2. Keep in dark on nutator for 20 mn. Dispose of Eppendorf tip in bucket containing a 10% bleach solution in order to decontaminate DAB.
4. Meanwhile prepare a 0.3% stock H_2_O_2_ in dH_2_O on ice.
5. Add 50μl stock H_2_O_2_ to vial containing embryos in DAB. Keep in dark. After 1-2 minutes, monitor reaction under microscope.

### Part 4: Embryo processing for photography and histology

1. When color reaction is complete, dispose of DAB in bleach bucket. Replace with 5 ml tap water.
2. Perform 2 10-minute washes in PBS.
3. To process for photography and wax sectioning, dehydrate in an ethanol series (25%, 50%, 75% and 100%) for 10 minutes each.
4. Replace the ethanol with 0.5 - 1 ml cedar wood oil (this will make the embryo translucent). Process for photography in cedar wood oil.
5. Following photography, return embryo to vial and replace the cedar wood oil with 100% ethanol. Repeat ethanol step.
6. Replace ethanol with 5% Fast green FCF in 100% ethanol, and incubate for 3 minutes (this step will make embryos visible for histology). Replace Fast green FCF solution with 100% ethanol and process for histology.

### Representative Results:


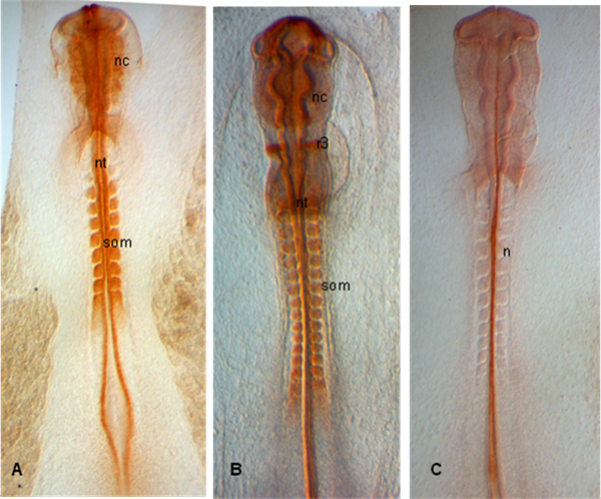


In the examples shown below, embryos are dissected at stages HH 10 (A), 12 (B) and 11 (C); Embryos show expression of PAX 7 in emerging neural crestas well as in somites and neural tube (A,B); In (C), notochord is labelled with 15.3B9 (Not-1) (Antibodies provided by Developmental Studies Hybridoma Bank).

## Discussion

This video demonstrates the different steps in performing whole-mount antibody staining in young chick embryos. This protocol is essentially used for the spatial and temporal characterization of novel antibodies in chick ^2,3^, as well as for the use of known antigenic markers to determine embryonic malformations following insult ^4^.
